# An Interesting Case of Acquired Hemophilia A in an Elderly Patient Presenting with Hematuria

**DOI:** 10.7759/cureus.6540

**Published:** 2020-01-02

**Authors:** Parth Mehta, Anil Kumar Reddy Reddivari

**Affiliations:** 1 Internal Medicine, University of Illinois College of Medicine, Peoria, USA

**Keywords:** factor viii, factor viii inhibitor, auto-antibodies, hemophilia a

## Abstract

A 90-year-old male with a past medical history of hypertension, chronic kidney disease stage II, and hyperlipidemia presented with complaints of intermittent hematuria. He had no prior history of hematuria or mucosal bleeds and denied having any trauma. His activated partial thromboplastin time (aPTT) was found to be mildly prolonged at 48.4 seconds and his factor VIII level was found to be very low at less than 3%.

## Introduction

Acquired hemophilia A (AHA) is a rare disease that results from the development of autoantibodies against factor VIII. These autoantibodies are called "inhibitors" and can lead to bleeding which sometimes can be severe. Bleeding can be spontaneous or post-traumatic in nature. Bleeding can be superficial like mucosal or subcutaneous or can be deep like intra-articular or intra-abdominal. It usually occurs in elderly patients with comorbidities and can be associated with underlying conditions such as diabetes, infections, malignancies or autoimmune diseases; however, in about half of the cases, it can be idiopathic. The primary goal of treatment includes hemostasis followed by eradication of the inhibitors. Management can be difficult and mortality risk remains high due to underlying comorbidities, bleeding, and complications associated with the treatment. The disease affects 1 to 1.5 per one million people annually and is likely underdiagnosed or misdiagnosed [1-2]. We report the case of an elderly male with AHA presenting as hematuria.

## Case presentation

A 90-year-old male with a past medical history of hypertension, chronic kidney disease stage II, and hyperlipidemia presented with complaints of intermittent hematuria. He had no prior history of mucosal bleeds and denied having any trauma. He denied having any history of easy bruisability. He had no pain. He has no prior history of hematuria and did not have any prostate issues. He had a history of cholecystectomy and left hip replacement. He had no current or past history of smoking, illicit drug use, or alcohol use. He had no history of taking any herbal or traditional medications. He did not have any significant medical issues in his family and family history was negative for any cancers or bleeding disorders. His temperature was 98.6° F, blood pressure 134/87 mmHg, pulse 83/minute, and respirations 14/minute. Physical examination was unremarkable for any acute findings.

Initial workup revealed hemoglobin (Hb) of 8.9 g/dl, hematocrit (Hct) of 27.1%, white blood cell count (WBC) of 9.4 10*3/uL, and platelet count of 235 10*3/uL. The metabolic panel was unremarkable and revealed electrolytes and liver function tests within the normal range. The patient's BUN and creatinine were 58 mg/dl and 1.3 mg/dl respectively which were also at baseline for him. His activated partial thromboplastin time (aPTT) was found to be mildly prolonged at 48.4 seconds. But prothrombin time (PT) was 11 seconds and international normalized ratio (INR) of 1.1, both within normal limits. The prostate-specific antigen was checked and came back at 1.2 ng/ml. Urinalysis was negative for nitrites, leukocyte esterase, and bacteria and showed only 0-1 white blood cells but showed a large amount of blood with more than 100 red blood cells. Peripheral smear was done which showed normocytic, normochromic anemia with mild anisocytosis. White blood cells and platelets showed no abnormality (Table 1).

**Table 1:** Initial lab values upon presentation aPTT: activated partial thromboplastin time; PT: prothrombin time; INR: international normalized ratio.

Test	Results	Reference value
Hemoglobin (g/dl)	8.9	13-17
Hematocrit (%)	27.1	39-49
White blood cells (10*3/uL)	9.4	3.60 - 9.50
Platelets (10*3/uL)	235	150 - 440
aPTT (seconds)	48.4	28-38
PT (seconds)	11	8.5-11.5
INR	1.1	0.9-1.2
Factor VIII (%)	<3	50-150
Factor VIII inhibitor titer (BU/ml)	12	Negative
Blood urea nitrogen (mg/dl)	58	10-25
Creatinine (mg/dl)	1.3	0.6-1.2
Prostate specific antigen (ng/ml)	1.2	0.7-3

**Figure 1:**
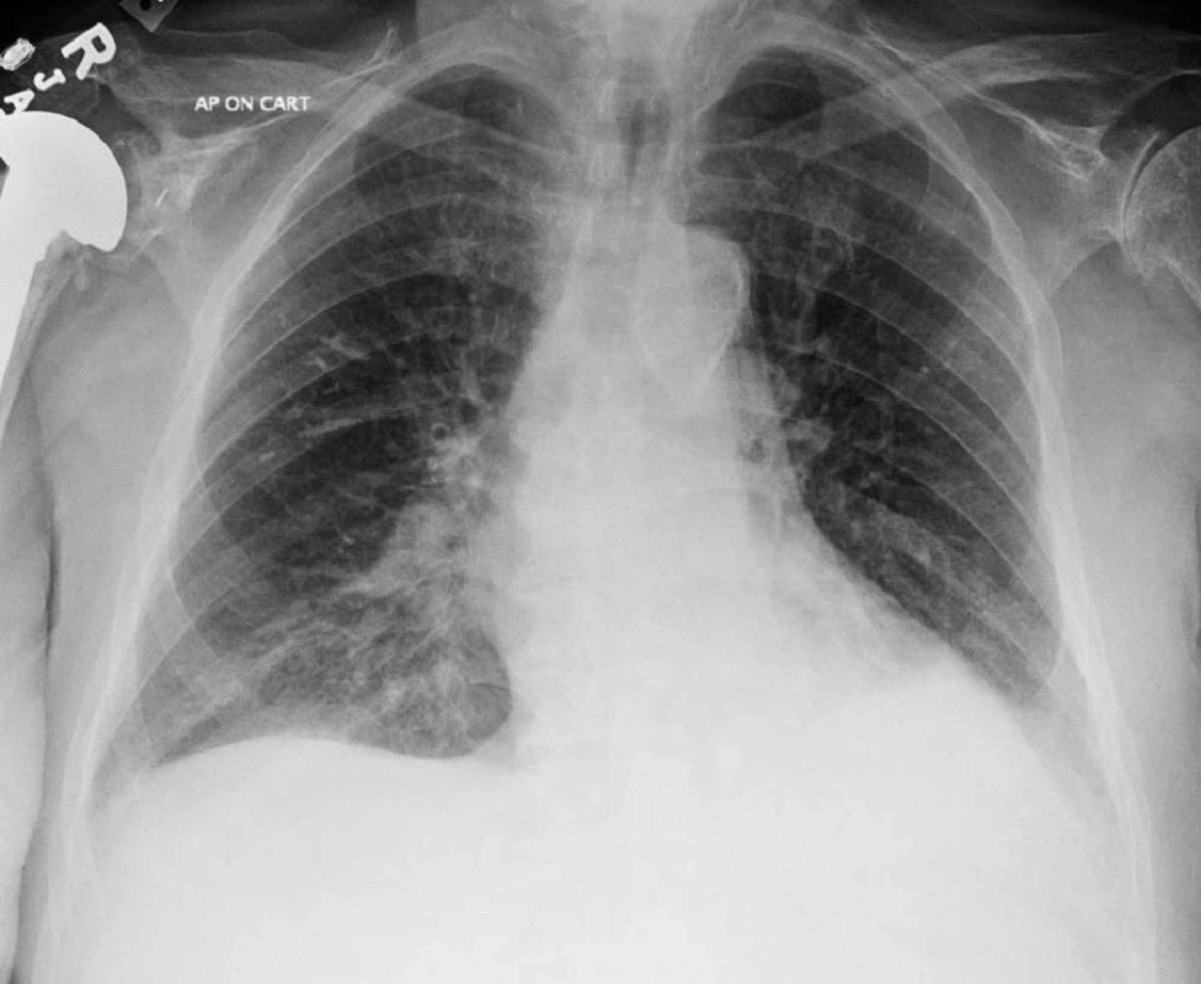
Chest X-ray was negative for any acute findings

A chest X-ray was done as a part of the routine investigations and returned normal (Figure 1).

**Figure 2:**
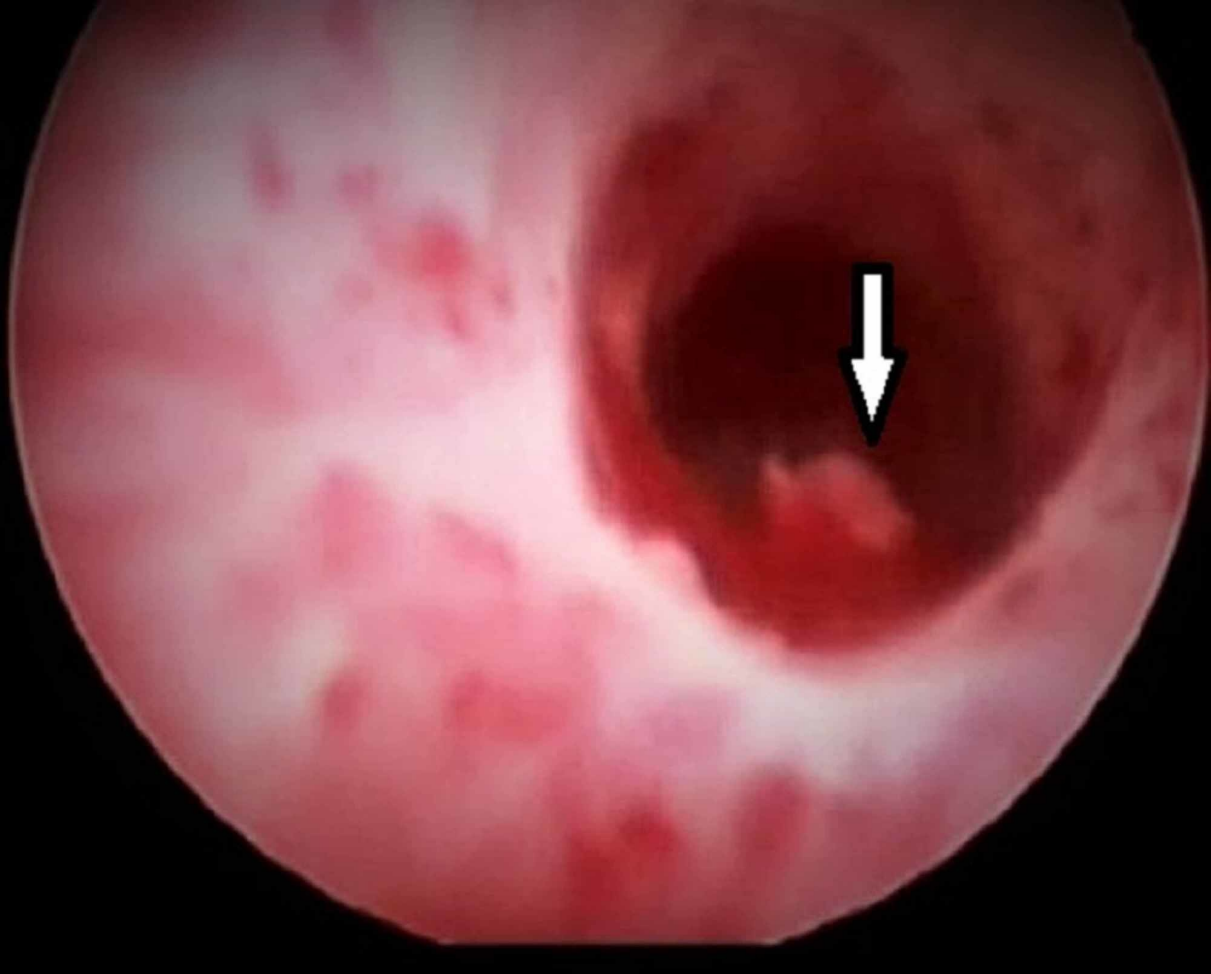
A small blood clot seen in the urinary bladder during cystoscopy

The patient was admitted with urology consultation and underwent a cystoscopy where no active bleeding was found and a small clot in the urinary bladder was evacuated (Figure 2). The patient then stopped bleeding for 24 hours but then again started having hematuria again which was more severe this time compared to the time of admission. Also, he started bleeding from the right arm where he had an intravenous line that had been placed earlier. 

At that time, Hematology consultation was obtained; aPTT was repeated which was mildly prolonged at 44.8 seconds. Factor VIII and Factor IX assays were performed and showed results of less than 3% (<3%) and 27% respectively. Factor VIII inhibitor levels were 12 BU/ml per Bethesda assay. His Hb was repeated which came back at 7.0. He was transfused with two units of blood. 

During this admission, he was started on corticosteroids with methylprednisolone being given intravenously initially at 80 mg followed by a prednisone tablet at 40 mg. The patient was also started on folic acid 1 mg and ferrous sulfate at 325 mg. The patient was simultaneously started on recombinant porcine factor VIII intravenously with 200 units/kg. His hematuria and bleeding from the right arm subsided within 24 hours. Factor VIII assay came back at 258%.

He was discharged to an inpatient rehabilitation unit. However, after five days in rehabilitation, he had a recurrence of hematuria. Factor VIII assay was done again and values came back at 140%. This was followed by a recurrence of bleeding from the right arm on the next day. At this time, the patient was readmitted to the medical floor from the rehabilitation unit. Factor VIII assay was done again and came back with a value of 9%. Factor VIII inhibitor levels were checked again and returned at 12 BU/ml per Bethesda assay again. 

The attempts to rid the patient of neutralizing antibodies to factor VIII with corticosteroids were only partially and temporarily successful. Unfortunately, even after a full recovery initially with recombinant porcine factor VIII, hematuria recurred and factor VIII levels were down again to 9%. This suggested that anti-factor VIII antibodies were now cross-reacting with recombinant porcine factor VIII. The patient was then started on recombinant factor VIIa as a bypassing agent at 90 mcg/kg and was repeated every three hours. Hemostasis was achieved at 24 hours and the patient did not have any further recurrence of hematuria after 48 hours after which he was discharged home with a follow-up appointment with Hematology as an outpatient. The patient was later on readmitted with pneumonia after two months but did not have any bleeding event at that time and his aPTT was 36.3. At a six-month follow-up, he had no more bleeding event and remained stable with aPTT of 35.9.

## Discussion

AHA is a rare disorder. It is caused by autoantibodies (inhibitors) that develop against endogenous factor VIII. These antibodies act against factor VIII and render it inactive which can cause bleeding that can be spontaneous or post-traumatic. It usually occurs in elderly patients who have underlying comorbidities like malignancy, autoimmune disorders, etc.; however, in about half of the cases, it can be idiopathic. AHA carries a high mortality risk due to underlying co-morbidities, bleeding, and complications from treatment. Disease affected 1 to 1.5 per one million people annually. It affects males and females equally. While most cases are reported in adults and mainly elderly people with the median age of 60 to 67 years, there have been cases reported ranging from two years to 89 years of age also. A small population of women can develop factor VIII inhibitors during pregnancy [3-4].

Many cases of AHA are idiopathic and no underlying cause is found like in the case of our patient who had no underlying comorbidities or conditions. The patient also had a thorough workup done and we were not able to find any underlying cause for his acquired hemophilia neither on physical examination nor on investigations. 

In acquired hemophilia, the bleeding pattern is different compared to that observed in congenital hemophilia A. While bleeding mainly occurs in the joints in congenital hemophilia A, bleeding occurs more commonly in the skin, soft-tissues, and mucosal lining. AHA can cause life-threatening bleeding and can cause severe complications related to bleeding and these episodes can be more severe compared to bleeding episodes in congenital hemophilia with comparable factor VIIIl levels. Patients can have epistaxis, gastrointestinal bleeding, skin and muscle hematomas, retroperitoneal bleed or hematuria which our patient had. Intracerebral bleeds can be more frequent than joint bleeding. Spontaneous bleeding (77.4%) is more common in AHA and less than 10% of bleeding episodes were associated with trauma, surgery or pregnancy [5]. 

Factor VIII activity level or strength of the inhibitor does not have any relationship with the severity of bleeding. Levels can be severely low (<1% of normal) but can be higher also within a safe range. Usually, the median titer of the antibodies in patients who develop bleeding is around 20 BU/ml but patients can present with high titers or even barely detectable titers. Also, there is no correlation between aPTT levels and the severity of bleeding in acquired hemophilia. Based on the review of patients from the European Acquired Hemophilia Registry (EACH2), there were about 48 patients diagnosed with acquired hemophilia based on prolonged aPTT out of which 33 patients had no bleeding event reported, and in 15 patients bleeding event occurred after diagnosis. This is different from congenital hemophilia where lower levels of the factor are associated with higher frequency and severity of bleeding [6]. 

Acquired hemophilia should be suspected when a new and unexpected bleeding event occurs in a patient without any personal or family history of bleeding. This is especially important in elderly patients with comorbidities or postpartum patients. It is important to diagnose acquired hemophilia in a timely fashion as bleeding events can be severe and can be fatal sometimes. Delay in diagnosis is possible as the disease is rare and due to a lack of familiarity with health care personnel. This can impact the management and prognosis of the patients. Extended coagulation testing should be done, if available, to rule out other causes of bleeding or aPTT prolongation. Many elderly patients may be on anticoagulation for venous thromboembolism or atrial fibrillation and the effects of anticoagulation agents in causing the bleeding should be ruled out first. Prolonged thrombin time and positive anti-Xa tests are signs of the effects of anticoagulation agents. After ruling out the effects of anticoagulation agents, extended coagulation testing should be performed if available mainly clotting factor activities of factor VIII and IX, aPTT and mixing studies for aPTT. Prolonged aPTT along with normal PT are the first-line tests for the diagnosis of AHA. Factor VIII levels are usually low but can be normal also. Very low levels are associated with longer remission times and poor prognosis. Bethesda assay can be done to quantify the anti-factor VIII antibodies or inhibitors [7-10].

Our patient had a mildly prolonged aPTT at 48.4 seconds and even after repeating it was 44.8 seconds. Mixing study is performed usually where the patient's plasma is mixed with pooled normal plasma in a ratio of 1:1. Patients with inhibitors to factor VIII in their plasm will end up inhibiting factor VIII in the normal plasma as well and measured aPTT will not be corrected. If the normal plasma cannot correct the aPTT by more than 50%, then the presence of inhibitors is confirmed. This is usually further evaluated by Bethesda assay. The mixing study was not performed in our hospital due to unavailability but Bethesda assay was performed which showed factor VIII inhibitor levels at 12 BU/ml [6]. Sometimes, prolonged aPTT with a normal PT can also be caused by the presence of a lupus anticoagulant but those patients typically present with thrombosis and not bleeding events, therefore, it was not done as it was not relevant in our patient due to his presentation with hematuria [11]. 

The most important goal of treatment in patients with acquired hemophilia is to achieve hemostasis, followed by medications to eradicate inhibitors and treating underlying comorbidities. In our patient, corticosteroids were given which was only partially and temporarily successful. This was followed by the administration of recombinant porcine factor VIII which was also temporarily successful as the patient had a recurrence of hematuria which suggested that anti-factor VIII antibodies were now cross-reacting with recombinant porcine factor VIII. The patient was then started on recombinant factor VIIa as a bypassing agent and hemostasis was achieved. This is important because patients who have high factor VIII inhibitor titers (>5 BU/ml), even high doses of human factor VIII are ineffective as antibodies will immediately block the administered factor VIII and with porcine factor VIII; cross-reactivity is possible and therefore the efficacy is unpredictable. This is exactly what we saw in our patient where he received recombinant porcine factor VIII after which factor VIII activity levels went up but later on, it started declining with a recurrence of hematuria from 164% to 9%. Due to these issues, bypassing agents such as recombinant factor VIIa (NovoSeven) which was used in our patient, or the activated prothrombin complex concentrate (aPCC) remain the first-line treatment. Recombinant factor VIIa activates factor X directly, without needing factor VIII or IX, to control the bleeding. However, conventional laboratory assays cannot measure this medication and the clinical response of the patients to the bleeding, after administration of the medication, remains the only way to assess the efficacy of the treatment. However, the medication is expensive and not without side effects and can cause thromboembolism in elderly patients with comorbidities [12].

Also, per the Journal of European Hematology Association, patients with more than 5 BU/ml titers of inhibitors should receive immunosuppressive therapy to eradicate inhibitors but its efficacy is limited in patients with inhibitor titers of more than 200 BU/ml. Our patient also received corticosteroid therapy to eradicate inhibitors but it was only successful partially and temporarily. Apart from corticosteroids, other immunosuppressive therapy agents include cyclophosphamide, azathioprine, 6-mercaptopurine, vincristine, rituximab, and cyclosporin A but the most commonly used therapy includes corticosteroids alone or a combination of corticosteroids and cyclophosphamide [13-14]. 

There are newer non-factor therapies that are being studied at this time. These include medications like emicizumab and anti-tissue-factor pathway inhibitor therapy such as concizumab and fitusiran. Emicizumab is a factor VIIIa mimetic antibody and targets both factor IXa and X, leading to faster activation of factor X and acts like factor VIIIa. It is already approved for patients with hemophilia A who have inhibitors [15]. Medications like concizumab work by modulating thrombin generation and downregulates the extrinsic pathway of blood coagulation while fitusiran works by inhibiting regulatory pathways by blocking antithrombin synthesis. These medications have been mostly studied in congenital hemophilia but are also promising to improve hemostasis in AHA patients in the future, however, more research is required to study them better [16-18].

## Conclusions

This case was unique as very few cases have been reported so far. The purpose of this case report was to create more awareness about the diagnosis and management options for acquired hemophilia as it can often go unrecognized or get misdiagnosed; a high index of suspicion is required when patients present with bleeding episodes in the absence of any underlying events or co-morbidities. In this patient, the cause of his acquired hemophilia is believed to be idiopathic as no identifiable underlying source causing the acquired hemophilia was found despite the workup. It has been reported that about 10% of patients who have acquired hemophilia have an underlying malignancy such as squamous cell cancer, chronic lymphocytic leukemia, non-Hodgkin's lymphoma, multiple myeloma, etc. Therefore, the patient should have a long-term follow-up and detailed malignancy workup as cases have been reported where AHA has been detected before the diagnosis of malignancy.
